# Evaluation of therapeutic effects of crocin in attenuating the progression of diabetic nephropathy: a preliminary randomized triple-blind placebo-controlled trial

**DOI:** 10.1186/s12906-022-03744-5

**Published:** 2022-10-08

**Authors:** Asma Jaafarinia, Behzad Kafami, Adeleh Sahebnasagh, Fatemeh Saghafi

**Affiliations:** 1 Department of nephrology, Shahid Rahnemoon hospital, Shahid Sadoughi University of Medical Sciences and Health Services, Yazd, Iran; 2 Diabetes Research Center, Shahid Sadoughi University of Medical Sciences and Health Services, Yazd, Iran; 3 Pharmaceutical Sciences Research Center, School of Pharmacy, Student Research Committee, Shahid Sadoughi University of Medical Sciences and Health Services, Yazd, Iran; 4 Department of Internal Medicine, Clinical Research Center, Faculty of Medicine, North Khorasan University of Medical Sciences, Bojnurd, Iran; 5 Department of Clinical Pharmacy, School of Pharmacy, Faculty of Pharmacy and Pharmaceutical Sciences Research Center, Shahid Sadoughi University of Medical Sciences and Health Services, Yazd, Iran

**Keywords:** Diabetes mellitus, Type 2; nephropathy, Crocin, Crocus

## Abstract

**Background:**

Diabetic nephropathy (DN) is one of the most important complications of type 2 diabetes (T2DM). Oxidative stress and inflammatory cytokines play an essential role in the development and progression of DN. Despite adopting appropriate therapies, many patients with DN progress to end-stage renal disease (ESRD). Therefore, exploring innovative strategies for better management of DN is crucial. Crocin, a natural compound found in saffron, has profound antioxidant, antifibrotic and anti-inflammatory properties. This study aimed to evaluate the therapeutic effects of crocin in attenuation of the progression of DN.

**Methods:**

In this randomized, triple-blind, placebo-controlled clinical trial, 44 patients with T2DM and microalbuminuria were randomly assigned to receive either crocin (15 mg/day) or a placebo for 90 days. Eventually, 40 patients completed the study: 21 patients in the crocin group and 19 in the placebo group. The primary outcome was a change in urine Albumin-to-Creatinine Ratio (uACR) from baseline to the end of the treatment period. We also evaluated metabolic, anthropometric, and biochemical parameters as the secondary outcomes.

**Results:**

The results of the present study showed that uACR increased in both groups, but the increment was not significantly higher in the crocin group compared with the placebo. Serum levels of transforming growth factor-β (TGF-β) decreased in the crocin group and increased in the placebo group, but none of these changes was significant. Crocin significantly reduced triglyceride (TG) as an important metabolic parameter (*P*-*Value* = 0.03).

**Conclusion:**

This study has shown that crocin may be a safe and potential adjunct to conventional therapies for DN patients but because of our limitations such as short duration of the treatment period, and prescribing low doses of crocin, we could not achieve the significant level.

## Introduction

DN is the main cause of the ESRD worldwide and affects 25–40% of diabetic patients with a higher prevalence in type 1 diabetes mellitus (T1DM) compared with T2DM [1]. It is characterized as an elevated urine albumin excretion associated with high blood pressure (BP). This leads to diminished estimated glomerular filtration rate (eGFR) and eventual kidney failure [2].

Various metabolic, inflammatory, and hemodynamic pathways have been implicated in the development and progression of DN. Chronic hyperglycemia, hypertension, dyslipidemia, protein kinase C (PKC) activation, and polyol metabolism abnormalities are some of the pathological events involved in this. Moreover, the generation of advanced glycation end-product (AGEs), reactive oxygen species (ROS), and increased secretion of profibrotic cytokines, e.g., TGF-β and connective tissue growth factor (CTGF) are the most common related factors [2, 3].

DN is known as an independent risk factor for cardiovascular disorders (CVD). The mortality of CVD is mostly related to DN and microalbuminuria in diabetes patients aged less than 50 years [2–4]. Currently, available therapies including management of hyperglycemia, dyslipidemia, and BP, are not uniformly successful in preventing, reversing, or stabilizing renal injury in patients with diabetic nephropathy. Eventually, many of these patients need some form of renal replacement therapies even if they receive the best available nephroprotective medicines [5].

Other common alternative pathways contributing to DN pathogenesis are oxidative stress and inflammation [6, 7]. Previous studies reported promising results of therapeutic agents that target these pathways [7, 8]. Nevertheless, the endeavor to find novel antioxidant and anti-inflammatory agents that prevent the progression of DN while lacking major adverse effects continues [6, 7, 9].

*Crocus sativus L.*, commonly known as saffron contains several compounds such as crocin, which is the main antioxidant in saffron [10]. Crocin (crocetin di-gentiobiose ester) is a bioactive constituent in the flowers of the *Crocus* and *Gardenia* genus [11]. Crocin exerts potent antioxidant [12], anti-inflammatory [12], antihypertensive [13], and antifibrotic [14] properties. This natural product has been used for the management of kidney and urinary disorders in folk medicine [15].

In a recent preclinical study in rat animal model, crocin has improved impaired renal functions as shown by reduction in serum creatinine (SCr) levels, blood urea nitrogen (BUN) and proteinuria with concomitant increase in urinary clearance of creatinine in animal model of diabetic nephropathy. Furthermore, crocin decreased the serum lactate dehydrogenase (LDH) and kidney content of nitric oxide synthase (NOS), malondialdehyde (MDA), toll-like receptors 4 (TLR-4) and interleukin 6 (IL-6) significantly with a remarkable increase in the renal antioxidants such as superoxide dismutase (SOD), glutathione (GSH) and serum catalase activity. Moreover, crocin inhibited the progression of renal fibrosis [12].

However, although this herbal medicine has a high safety profile with no major side effects [16], no clinical trial to date has evaluated the potential supplemental efficacy of crocin in preventing the progression of DN. Considering these facts; the current trial was performed to evaluate the efficacy of the crocin in attenuating the progression of DN in patients with T2DM.

## Materials and methods

### Trial design

This study was a randomized, triple-blind, placebo-controlled, 2-arm parallel-group, phase 2 clinical trial using a 1:1 ratio of allocation. All methods were performed in accordance with relevant guidelines and regulations. This study was reviewed and approved by the local ethics committee of Yazd University of Medical Sciences (IR.SSU.MEDICINE.REC.1398.085) and performed in accordance with the Declaration of Helsinki and Good Clinical Practice Guidelines. The study was registered on Iranian Registry of Clinical Trials (IRCT20190810044500N4). All patients were given informed written consent before enrollment in this study.

### Participants

Patients aged ≥18 years with T2DM who received routine care of DM and were referred to a university affiliated Diabetes Clinic were evaluated for inclusion criteria eligibility. Baseline demographic and laboratory data were recorded.

Inclusion criteria were: (1) a history of T2DM for at least 5 years, as defined by the World Health Organization (WHO) (2) fasting uACR ≥30 mg/g on two separate occasions within the past 3 months (3) glycosylated hemoglobin A1c (HbA1c) < 8% (4) systolic blood pressure (SBP) < 160 or diastolic blood pressure (DBP) < 100 mmHg (5) SCr levels ≤2 mg/dL (6) treatment of hyperglycemia with (but not limited to) an oral hypoglycemic agent or insulin (7) treatment of hypercholesterolemia within (but not limited to) a statin.

Exclusion criteria were: (1) eGFR < 30 mL/min/1.73 m^2^ (2) chronic heart failure with New York Heart Association (NYHA) class III or IV (3) alcohol dependency or cigarette smoking (4) use of nonsteroidal anti-inflammatory drugs (NSAIDs) and antioxidant supplements within 3 months before enrollment in the study (5) recent stroke, active cancer or anemia (6) recurrent urinary tract infection (UTI) (7) pregnancy and lactation (8) drug intolerance (9) serious medical conditions in which the patient is unable to attend the follow-up visits (10) mental conditions that prevent informed consent for enrollment in the study or follow the study protocol.

### Interventions

Both crocin and placebo tablets were purchased from Pouyesh Darou Sina Pharmaceutical Company and were supplied by the Yazd School of Pharmacy in similar prepacked bottles numbered for each patient according to the randomization sequence. Patients received either one tablet of crocin 15 mg or one tablet of placebo daily for 90 days. This dosage was selected based on the results of previous studies and for an acceptable safety profile [17]. Patients then continued taking their antihypertensive, antidiabetic, and antidyslipidemic medications with no modification in either dosage or type of drug throughout the study. They were also advised to avoid taking any antioxidant or vitamin supplements and saffron in food during the study.

### Randomization and masking

Forty-four eligible patients were randomly assigned to either of two groups (crocin and placebo). A randomized list was generated by Microsoft Excel software with a block randomization method. Both intevention and placebo tablets were similar in size, shape, weight, and color. Clinical investigators, laboratory personnel, and patients were all masked to the treatment assignment. To ensure the adherence of the patients to their medications, the research team kept in close contact with all patients throughout the follow-up visits.

### Assessment

Demographic data including age, gender, height, weight, body mass index (BMI), and BP were measured and recorded for each patient. After 12 h of fasting, 15- ml blood and spot urine samples were collected at baseline and after 90 days of intervention for each patient. All blood samples were immediately centrifuged at 4000 rpm (rpm) for 10 minutes, and the sera were transferred to a − 80 °C freezer until biochemical analysis. Fresh, morning first-void urine samples were also collected for measurement of urine albumin and creatinine. at baseline and at the end of the treatment phase for all patients.

To decrease the variation in urinary albumin excretion, patients were instructed to maintain similar physical activities on each day before overnight urine sampling, and unusual changes in physical activity should be avoided.

Metabolic, anthropometric and biochemical parameters, including weight, height, BMI, BP, fasting blood sugar (FBS), HbA1c, erythrocyte sedimentation rate (ESR), C-reactive protein (CRP), BUN, urine microalbumin, SCr, uACR, eGFR, lipid profile, and serum TGF-β were evaluated at baseline and at the end of the treatment period in all patients. Height was measured with a stadiometer, and weight with a standard balance weighing scale. BMI was calculated as weight divided by the square of height (kg/m^2^). BP was measured two times on the right arm with a standard mercury sphygmomanometer (mmHg) after a 15-min rest in sitting position. Serum and urinary creatinine were measured by an autoanalyzer using the Jaffé method. Urinary albumin, FBS, lipid profiles, BUN, CRP were also measured by autoanalyzer. HbA1c was measured by high-performance liquid chromatography (HPLC). ESR was measured by an ESR analyzer using Westergren method. Serum TGF-β were measured by enzyme-linked immunosorbent assay (ELISA) kit (Karmania pars gene) and eGFR was calculated using the 4-variable modification of diet in renal disease (MDRD) Study equation.

As part of the safety evaluation, adverse effects of Crocin including sleep disorders, nausea/ vomiting, feet swelling, mild tremor, stomachache, increased appetite, redness, swelling/burning of eyes, and sub conjunctival hemorrhage were asked weekly.

### Sample size

Sample size was calculated by a statistician based on data from a previous study [18]. to detect a mean difference of 450 mg/g in absolute change in uACR between the 2 groups with standard deviation of 1000 mg/g, 2-sided significance level of 5%, and power of 80%, the sample size was calculated 22 patients per group.

$$n={\left({\textrm{Z}}_{\frac{\upalpha}{2}}+{\textrm{Z}}_{\upbeta}\right)}^2\ 2{S}^2/{\left({X}_1-{X}_2\right)}^2$$

### Statistical analysis

The quantitative and qualitative variables were reported as mean ± standard deviation (SD), median ± interquartile range (IQR), and number (frequency). The normality of the data was assessed by Shapiro-Wilk and Kolmogorov-Smirnov tests. Baseline data with normal distribution were compared between two groups using independent-samples *t*-test. Baseline data with skewed distribution were analyzed using Mann-Whitney *U* test. For comparisons of variables with normal and skewed distribution of data before and after the intervention in each group, paired-samples *t*-test and Wilcoxon Signed Ranks Test were performed, respectively. Chi-square test was used to compare qualitative variables. All the statistical analysis was conducted by Statistical package for social science (SPSS) software version 26. *P*-values < 0.05 were considered statistically significant.

## Results

**Fig. 1:**
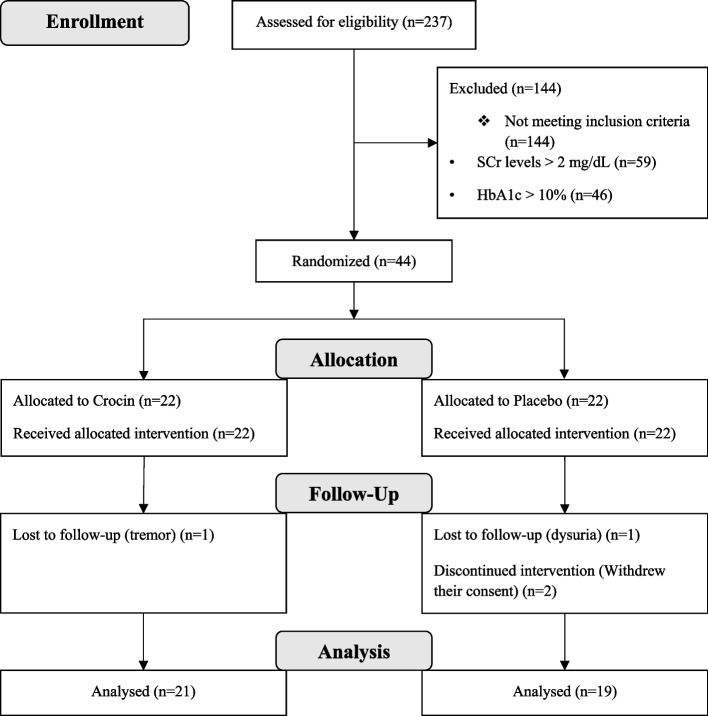
The Flowchart of the trial of Crocin vs placebo

The eligibility screening phase of this study began in December 2019 and was completed in November 2020. A total of 44 (52% men) were enrolled in the study and randomly assigned (1:1) to the crocin group (22 patients) or the placebo group (22 patients). Two patients in the placebo group withdrew their follow-up sections and dropped out of the study because they did not use the formulation correctly. Moreover, one patient in the crocin group were excluded from the study due to tremor and one patient in the placebo group due to dysuria, although none of these complaints were serious. Eventually, 40 patients completed the 3-month treatment phase of the study: 21 patients in the crocin group and 19 in the placebo group (Fig. 1). As illustrated in Table 1, there were no statistically significant differences between the crocin and placebo groups in terms of baseline anthropometric, clinical and biochemical parameters.

**Table 1:** Baseline Characteristics of Participants in Placebo and Crocin Groups

Parameters	Placebo group (No = 19)	Crocin group (No = 21)	*p-value*
Age^a^ (y)	62.68 ± 9.84	63.86 ± 10.62	0.72
Men, No (%)	11 (57.90)	12 (57.14)	0.96
Duration of diabetes^a^ (y)	11.11 ± 7.48	13.20 ± 3.27	0.28
BMI^a^ (kg/m^2^)	27.26 ± 3.34	27.21 ± 3.86	0.97
Blood Pressure^a^ (mmHg)			
SBP	148.20 ± 22.45	150.25 ± 18.08	0.31
DBP	75.33 ± 12.31	81.44 ± 16.46	0.25
Comorbidities, No (%)			
Migraine	0 (0.0)	1 (4.8)	0.33
Depression	1 (5.3)	0 (0.0)	0.29
Asthma	1 (5.3)	0 (0.0)	0.29
Dyslipidemia	16 (84.2)	19 (90.5)	0.55
Rheumatoid Arthritis	2 (10.5)	4 (19.0)	0.45
Hypothyroidism	0 (0.0)	2 (9.5)	0.17
Non-trial Medication Categories, No (%)			
ACEIs/ARB	17 (89.5)	19 (90.5)	0.92
β-blocker	7 (36.8)	8 (38.1)	0.93
CCB	6 (31.6)	5 (23.8)	0.58
Insulin	8 (42.1)	10 (47.6)	0.73
Biguanide	14 (73.7)	16 (76.2)	0.85
Sulfonylurea	7 (36.8)	9 (42.9)	0.70
Lipid-lowering Agent	16 (84.2)	19 (90.5)	0.55
Antiplatelet Agent	11 (57.9)	14 (66.7)	0.57
Others	13 (68.4)	10 (47.6)	0.58
Laboratory measurements at inclusion			
FBS^a^ (mg/dL)	136.58 ± 57.17	140.52 ± 36.71	0.79
Serum TC^a^ (mg/dL)	157.89 ± 58.85	133.24 ± 29.49	0.79
Serum TG^a^ (mg/dL)	143.52 ± 63.89	157.89 ± 84.69	0.55
Serum HDL-C^a^ (mg/dL)	48.68 ± 14.61	41.62 ± 9.93	0.08
Serum LDL-C^a^ (mg/dL)	79.21 ± 53.38	64.51 ± 23.44	0.26
BUN^a^ (mg/dL)	45.06 ± 12.34	47.32 ± 27.83	0.75
HbA1c^a^ (%)	7.32 ± 1.04	6.87 ± 0.67	0.11
SCr^b^ (mg/dL)	1.10 ± 0.40	1.20 ± 0.40	0.89
ESR^b^ (mm/hr)	17.50 ± 18.00	17.00 ± 28.00	0.92
CRP^b^ (mg/dL)	5.00 ± 0.00	5.00 ± 0.50	0.06
Urine micro albumin^b^ (mg/dL)	181.00 ± 318.80	185.0 ± 232.5	0.51
uACR^b^ (mg/g)	186.85 ± 240.8	161.00 ± 250.8	0.88
eGFR^b^ (mL/min/1.73 m2)	58.00 ± 39.25	61.00 ± 20.00	0.98
Serum TGF-β^2^ (ng/mL)	16.93 ± 1.98	17.10 ± 0.80	0.64

^a^Data are expressed as Mean ± SD

^b^Data are expressed as Median ± IQR, *No* Number, *y* Year, *BMI* Body Mass Index, *SBP* Systolic Blood Pressure, *DBP* Diastolic Blood Pressure, *ACEIs* Angiotensin-Converting Enzyme inhibitors, *ARB* Angiotensin II Receptor Blockers, *CCB* Calcium Channel Blockers, *FBS* Fasting Blood Sugar, *TC* Total Cholesterol; *TG* Triglyceride; *HDL-C* High Density Lipoprotein-Cholesterol, *LDL-C* Low Density Lipoprotein-Cholesterol, *BUN* Blood Urea Nitrogen, *HbA1c* Hemoglobin A1c, *SCr* Serum Creatinine, *ESR* Erythrocyte Sedimentation Rate, *CRP* C-Reactive Protein, *uACR* Urinary Albumin-Creatinine Ratio, *eGFR* estimated Glomerular Filtration Rate, *TGF-β* Transforming Growth Factor-β

As listed in Table 2, comparison of the crocin and placebo groups for changes in variables (before- after) showed that mean serum TG levels decreased significantly in the crocin group compared with the increasing values in the placebo group (*P-Value* = 0.03). Moreover, the mean BMI, SBP, and DBP and median serum TGF-β levels decreased in the crocin group compared with the placebo group but none of these changes were statistically significant. Mean FBS and HgA1c levels increased in both groups. Although this increase was greater in the placebo group, these changes were not significant.

**Table 2:** Comparison of Metabolic Profile, Clinical and Biochemical Variables Before and After 3 Months Crocin and Placebo Interventions

Parameters	Crocin group (No = 21)				Placebo group (No = 19)				Between group *p-value*
	Before	After	After-Before	Within group *p-value*	Before	After	After-Before	Within group *p-value*	
Data are expressed as Mean ± SD									
BMI (kg/m2)	27.21 ± 3.86	27.02 ± 3.95	−0.46 ± 1.25	0.12	27.26 ± 3.34	27.16 ± 3.44	−0.10 ± 0.56	0.49	0.30
SBP (mm Hg)	150.25 ± 18.08	143.13 ± 26.07	−7.12 ± 14.95	0.07	138.20 ± 42.45	141.13 ± 18.71	2.93 ± 38.35	0.77	0.34
DBP (mm Hg)	81.44 ± 16.46	77.19 ± 9.66	−4.25 ± 17.02	0.33	75.33 ± 12.31	78.13 ± 8.87	−2.80 ± 8.39	0.22	0.16
FBS (mg/dL)	140.52 ± 36.71	146.24 ± 49.62	5.71 ± 38.58	0.51	136.58 ± 57.17	159.21 ± 62.62	22.63 ± 54.04	0.08	0.26
Serum TC (mg/dL)	133.24 ± 29.49	141.81 ± 40.97	8.57 ± 33.22	0.25	157.89 ± 58.85	156.21 ± 64.61	−1.68 ± 22.33	0.75	0.26
Serum TG (mg/dL)	157.89 ± 84.69	133.79 ± 43.21	−24.10 ± 74.57	0.17	143.52 ± 63.89	169.33 ± 69.71	25.81 ± 61.48	0.06	0.03^*^
Serum HDL-C (mg/dL)	41.62 ± 9.93	45.95 ± 12.28	4.33 ± 14.36	0.18	48.68 ± 14.61	52.05 ± 14.41	3.37 ± 15.57	0.36	0.84
Serum LDL-C (mg/dL)	64.51 ± 23.44	63.95 ± 33.57	−0.56 ± 26.58	0.92	79.21 ± 53.38	71.89 ± 61.40	− 7.32 ± 26.41	0.24	0.43
BUN (mg/dL)	47.32 ± 27.83	46.65 ± 22.79	−0.67 ± 16.79	0.86	45.06 ± 12.34	43.78 ± 13.01	−1.28 ± 10.78	0.62	0.90
HbA1c (%)	6.87 ± 0.67	7.03 ± 0.83	0.16 ± 0.56	0.22	7.32 ± 1.04	7.87 ± 1.69	0.48 ± 0.98	0.05	0.31
Data are expressed as Median ± IQR									
SCr (mg/dL)	1.20 ± 0.40	1.10 ± 0.50	0.00 ± 0.30	0.41	1.10 ± 0.40	1.10 ± 0.70	0.00 ± 0.22	0.75	0.59
ESR (mm/hr)	17.00 ± 28.00	22.00 ± 11.00	1.00 ± 15.50	0.98	17.50 ± 18.00	14.50 ± 17.00	−2.50 ± 7.75	0.05	0.15
CRP (mg/dL)	5.00 ± 0.50	5.00 ± 1.00	0.00 ± 1.50	0.79	5.00 ± 0.00	5.00 ± 0.00	0.00 ± 0.00	0.46	0.94
Urine micro albumin (mg/dL)	185.0 ± 232.5	128.5 ± 287.4	34 ± 141.55	0.26	181.00 ± 316.80	141.00 ± 131.3	11.50 ± 248.75	0.98	0.718
UACR (mg/g)	161.00 ± 250.8	152.5 ± 188.8	10.5 ± 151	0.46	186.85 ± 240.8	157.5 ± 243.8	16 ± 143	0.58	0.98
eGFR (mL/min/1.73 m^2^)	61.00 ± 20.00	67.00 ± 36.00	0.00 ± 22.50	0.13	58.00 ± 39.25	62.00 ± 44.75	0.00 ± 9.75	0.94	0.47
Serum TGF-β (ng/mL)	17.10 ± 0.80	16.92 ± 0.85	−0.21 ± 1.21	0.56	16.93 ± 1.98	17.29 ± 0.94	0.33 ± 2.40	0.35	0.21

*No* Number, *SD* Standard Deviation, *BMI* Body Mass Index, *SBP* Systolic Blood Pressure, *DBP* Diastolic Blood Pressure, *FBS* Fasting Blood Sugar, *TC* Total Cholesterol, *TG* Triglyceride, *HDL-C* High Density Lipoprotein-Cholesterol, *LDL-C* Low Density Lipoprotein-Cholesterol, *BUN* Blood Urea Nitrogen, *HbA1c* Hemoglobin A1c, *SCr* Serum Creatinine, *IQR* Interquartile Range, *ESR* Erythrocyte Sedimentation Rate, *CRP* C-Reactive Protein, *uACR* Urinary Albumin-Creatinine Ratio, *eGFR* Estimated Glomerular Filtration Rate, *TGF-β* Transforming Growth Factor-β; *Statistically significant (*p*-value < 0.05)

Mean values for changes in TC, HDL, LDL, BUN, and as well as median values for changes in SCr, ESR, CRP, urine microalbumin, uACR, and eGFR were not significantly different between the two groups. Mild adverse effects in the form of tremor (1 of 22 patients) and dysuria (1 of 22 patients) were observed in participants treated with crocin and placebo tablets, respectively. Notably, the crocin tablet caused no significant side effect.

## Discussion

In this randomized clinical trial, we investigated the potential nephroprotective effect of crocin on microalbuminuria, and its effect on biomarkers of inflammation, and lipid profile in patients with DN for the first time. The results of the present study demonstrated that consumption of crocin 15 mg daily for 90 days results in decrease in BMI, SBP, DBP, serum TG, and serum TGF-β levels, but only changes in serum TG levels were statistically significant.

DN is one of the serious complications of T2DM and a major cause of ESRD. Urinary albumin levels reflect the degree of renal damage. It is an independent risk factor for the progression of renal disease to ESRD. An increasing number of clinical studies have found a strong relationship between the rate of albuminuria and the decline in eGFR [19–22]. As a result, urine albumin is an important prognostic marker and its drop is a goal of treatment in DN.

Previous evidence suggested that immunological and inflammatory processes play a key role in the development and progression of DN. Inflammatory cytokines such as IL-1, IL-6, IL-18, Tumor necrosis factor-α (TNF-α), TGF-β, and Monocyte Chemoattractant Protein-1 (MCP-1) have involved in the pathophysiology of DN [23, 24]. Furthermore, high blood glucose levels, abnormal hemodynamics, immune and inflammatory responses, and damage to the glomerular basement membrane and podocytes are the pathophysiological basis of proteinuria. In addition to tight glycemic control, angiotensin receptor blockers (ARBs) are effective interventions in DN [19, 20, 25–27]. The efficacy of ARBs is likely due to their ability to block RAS, lower blood pressure, suppress the expression of certain inflammatory cytokines and protect podocytes [28–30]. Considering the side effects of these drugs, such as hyperkalemia and an increase in SCr, they are not effective in all patients, at least not at the doses currently prescribed. Therefore, it is necessary to develop new strategies for DN patients with proteinuria.

Preclinical studies on animal models have shown that crocin can decrease albuminuria and other indicators of renal damage by decreasing inflammation and biomarkers of oxidative stress [12, 31]. However, there are no reports of clinical trials examining the effects of crocin on DN.

Dyslipidemia is a risk factor for the progression of DN through the development of glomerulosclerosis and tubulointerstitial lesions with concomitant accelerated atherosclerosis [32]. Clinical studies have suggested that TG promotes glomerular and tubulointerstitial injury via mediators such as ROS, chemokines, cytokines, TGF-ß1 gene expression, and macrophage extravasations into the glomeruli and tubules [32, 33]. lipid-lowering therapy reduces the incidence of macrovascular complications and microvascular complications including retinopathy, nephropathy, and autonomic neuropathy [34]. Our results showed that the effect of crocin on lipid profiles was significant in reduction of TG levels with no significant change in TC, HDL and LDL. These findings are consistent with the results of a clinical trial that assessed the effect of crocin on lipid profile in patients with non-alcoholic fatty liver disease (NAFLD). They found that consumption of crocin for 8 weeks could significantly reduce TG levels in comparison to the placebo. Howevere, there was no significant difference in the levels of LDL and HDL between crocin and placebo [35]. In a preclinical study in a rat animal model investigating the hypolipidemic mechanism of crocin in rats, this natural chemical selectively inhibited the activity of pancreatic lipase. Crocin also inhibited the absorption of fat and cholesterol and enhanced their fecal excretion [36].

TGF-β is a cytokine that plays an essential role in the pathogenesis of DN by mediating glomerulosclerosis and tubulointerstitial fibrosis [5, 8]. TGF-β urinary and serum levels are elevated in DN patients and correlate directly with the development of proteinuria and progression of DN [37, 38]. There are reports of some other novel candidates that have reduced proteinuria in DN which have also reduced TGF-β levels [39, 40]. Some clinical trials have shown that herbal medicines like curcumin and silymarin can reduce serum and urinary levels of TGF-β in DN patients. Preclinical studies have also reported the anti-TGF-β effects of crocin [12, 14]. Moreover, in a recent preclinical study, crocetin Nano-particles, a metabolite of crocin, significantly altered the expression TGF-β in animal model of DN. However, until now, no studies have been performed to determine the effect of crocin on TGF-β levels in humans. Although in this study, crocin reduced the serum levels of TGF-β as an important indicator in the progression of diabetic nephropathy, but this decrease was not statistically significant. Perhaps longer duration of intervention and follow-up periods could make changes in serum levels of this inflammatory marker of the kidney significant.

So far, many clinical studies have been performed to determine the supplemental effect of herbal compounds to common therapies for DN, and some of them, such as resveratrol, curcumin, and silymarin, have shown primary beneficial effects on biomarkers of oxidative stress and metabolic parameters [18, 41]. Considering the high safety profile of herbal medicines with no major side effects along with their contribution in various pathways involved in the pathogenesis and progression of DN [18, 40, 41], supplementing diabetic patients with herbal medicines could be a potential therapeutic strategy for improvement of diabetic nephropathy.

The main limitations to our study were short fallow-up period, and only one low dose of crocin was evaluated. In addition, early predictors for acute kidney injury (AKI) like neutrophil gelatinase-associated lipocalin (NGAL) was not measured. This was a preliminary study in which the effect of crocin on DN was evaluated for the first time. Further studies are required to assess both the longer-term efficacy and safety of crocin.

## Conclusion

To our knowledge, this is the first randomized, triple-blind, placebo-controlled, clinical trial that evaluated the possible efficacy and safety of crocin in attenuating the proteinuria in T2DM patients with DN. Due to the anti-inflammatory, antioxidative and antifibrotic properties of crocin as well as the positive effects of crocin in improving diabetic nephropathy in an animal studies, we conducted the present study. However, we could not find any statistically significant change in any measured parameters except TG. Future clinical trials with larger sample size, longer durations of intervention, and higher doses of crocin as well as measurement of specific biomarkers of renal injury will determine the efficacy, safety, and possible underlying mechanisms of action of crocin in attenuation of the progression of diabetic nephropathy.

## Data Availability

All data generated or analyzed during this study are included in this published article.

## Abbreviations

DN: Diabetic nephropathy
T2DM: Type 2 diabetes mellitus
RAS: Renin-Angiotensin System
ESRD: End-stage renal disease
uACR: Urine albumin to creatinine ratio
TGF-β: Transforming growth factor-β
TG: Triglycerides
T1DM: Type 1 diabetes mellitus
BP: Blood pressure
eGFR: Estimated glomerular filtration rate
PKC: Protein kinase C
AGEs: Advanced glycation end-product
ROS: Reactive oxygen species
CTGF: Connective tissue growth factor
CVD: Cardiovascular disorders
SCr: Serum creatinine
BUN: Blood urea nitrogen
LDH: Lactate dehydrogenase
NOS: Nitric oxide synthase
MDA: Malondialdehyde
TLR-4: Toll-like receptors 4
IL-6: Interleukin 6
SOD: Superoxide dismutase
GSH: Glutathione
WHO: World health organization
HbA1c: Glycosylated hemoglobin A1c
SBP: Systolic blood pressure
DBP: Diastolic blood pressure
NYHA: New York heart association
NSAID: Nonsteroidal anti-inflammatory drug
UTI: Urinary tract infection
BMI: Body mass index
rpm: Revolutions per minute
FBS: Fasting blood sugar
ESR: Erythrocyte sedimentation rate
CRP: C-reactive protein
TC: Total cholesterol
LDL: Low-density lipoprotein
HDL: High-density lipoprotein
HPLC: High-performance liquid chromatography
ELISA: Enzyme-linked immunosorbent assay
MDRD: Modification of diet in renal disease
SD: Standard deviation
IQR: Interquartile range
SPSS: Statistical package for social science
TNF-α: Tumor necrosis factor-α
ARB: Angiotensin Receptor Blocker
ACEIs: Angiotensin-Converting Enzyme inhibitors
CCB: Calcium Channel Blockers
AKI: Acute kidney injury
NGAL: Neutrophil gelatinase-associated lipocalin
MCP-1: Monocyte Chemoattractant Protein-1
NAFLD: Non-alcoholic fatty liver disease
Kg/m^2^: Kilogram per Squared Meter
mmhg: millimetre(s) of mercury
IRCT: Iranian Registry of Clinical Trials
